# Recombinant Dengue virus protein NS2B alters membrane permeability in different membrane models

**DOI:** 10.1186/s12985-015-0456-4

**Published:** 2016-01-04

**Authors:** Moisés León-Juárez, Macario Martínez-Castillo, Gaurav Shrivastava, Julio García-Cordero, Nicolás Villegas-Sepulveda, Mónica Mondragón-Castelán, Ricardo Mondragón-Flores, Leticia Cedillo-Barrón

**Affiliations:** Departmento de Biomedicina Molecular, Centro de Investigacion y Estudios avanzados IPN, Av. Instituto Politecnico 2508 Col. San Pedro Zacatenco, 07360 México, Mexico; Departamento de Bioquímica, CINVESTAV IPN Av, IPN # 2508 Col. San Pedro Zacatenco, 07360 México, DF Mexico; Present Address: Departamento de Inmunobioquimica, Instituto Nacional de Perinatología, Montes Urales #800 Col. Lomas de Virreyes, 1100 México, Mexico

**Keywords:** *Dengue virus*, NS2B, Membrane permeability, Virus, Viroporin, Oligomerize, *Flavivirus*

## Abstract

**Background:**

One of the main phenomena occurring in cellular membranes during virus infection is a change in membrane permeability. It has been observed that numerous viral proteins can oligomerize and form structures known as viroporins that alter the permeability of membranes. Previous findings have identified such proteins in cells infected with Japanese encephalitis virus (JEV), a member of the same family that Dengue virus (DENV) belongs to (*Flaviviridae*). In the present work, we investigated whether the small hydrophobic DENV protein NS2B serves a viroporin function.

**Methods:**

We cloned the DENV NS2B sequence and expressed it in a bacterial expression system. Subsequently, we evaluated the effect of DENV NS2B on membranes when NS2B was overexpressed, measured bacterial growth restriction, and evaluated changes of permeability to hygromycin. The NS2B protein was purified by affinity chromatography, and crosslinking assays were performed to determine the presence of oligomers. Hemolysis assays and transmission electron microscopy were performed to identify structures involved in permeability changes.

**Results:**

The DENV-2 NS2B protein showed similitude with the JEV viroporin. The DENV-2 NS2B protein possessed the ability to change the membrane permeability in bacteria, to restrict bacterial cell growth, and to enable membrane permeability to hygromycin B. The NS2B protein formed trimers that could participate in cell lysis and generate organized structures on eukaryotes membranes.

**Conclusions:**

Our data suggest that the DENV-2 NS2B viral protein is capable of oligomerizing and organizing to form pore-like structures in different lipid environments, thereby modifying the permeability of cell membranes.

## Background

Dengue viruses (DENVs) are enveloped (+) sense RNA viruses belonging to the *Flaviviridae* family. The DENV replication cycle initiates following receptor binding to the host cell membrane, which is followed by internalization via endocytosis and subsequent release into the cytoplasm. The genome is then translated into a large polyprotein, which is proteolytically processed to yield 3 structural proteins (envelope, membrane precursor, and capsid) and 7 non-structural (NS) proteins (NS1, NS2A, NS2B, NS3, NS4A, NS4B, and NS5) [1, 2].

Cells infected with DENV undergo a series of detrimental functional and structural changes, such as cell rounding, shrinkage, and dislodgment from the growth surface [3]. In addition, the membranes of DENV-infected cells are compromised during viral RNA replication, assembly, and egress, which induce remodeling and redistribution of distinct cell membrane structures [4, 5] including the rough endoplasmic reticulum (ER) and Golgi apparatus [6]. These phenomena result in dramatic cytopathic effects that compromise the viability of infected cells.

Results from several studies have shown that in addition to cytopathic effects, another common feature of infected cells is the modification of host cell membrane permeability resulting from the incorporation of viral proteins into the infected cell membrane. This group of viral proteins is collectively referred to as viroporins [7–9], which are small hydrophobic viral proteins that oligomerize in the membranes of different intracellular compartments and cause cell permeabilization [10].

All viroporins share structural motifs, such as hydrophobic domains that form an amphipathic α-helix, and a cluster of basic residues, which can interact with negatively charged lipids [11]. Viroporins may alter membrane permeability to facilitate different replication steps, such as viral entry and egress. While these molecules may be nonessential for viral genome replication, evidence suggests that they are required for the production of infective particles [12, 13] In addition, viroporins influence several cellular functions, including vesicular trafficking [14], membrane remodeling [15], ion homeostasis [16], apoptosis induction [17], and activation of inflammatory mechanisms that may participate in pathogenesis [18].

Viroporins have been identified in several RNA viruses including *Flaviviridae* family members such as hepatitis C and Japanese encephalitis virus (JEV). Results from a study [19] showed that small hydrophobic nonstructural JEV proteins can influence membrane permeability. In that study, the JEV protein NS2B was found to have membrane-destabilizing activity (MDA) in all assays evaluated [19]. Furthermore, DENV also expresses the NS2B protein, and the NS2B proteins of JEV and DENV show conserved structural and functional characteristics. These characteristics include the hydrophilic segment, which is required for the cofactor activity of viral protease NS3, and the 3 hydrophobic regions that are thought to be responsible for membrane association and to generate the MDA. In addition, we previously demonstrated that NS2B is localized in cellular membranes (particularly in lipid rafts) due to the hydrophobic regions [20]. Data from a subsequent study suggested that the NS2B protein contains alpha-helical transmembrane domains that direct folds within micelles, indicating the ability of this protein to associate with membranes [21]. These lines of evidences suggest the possibility that NS2B of DENV may exert a function that is analogous to NS2B of JEV.

In the present study, we observed by in silico analysis that the NS2B has a highly hydrophobic profile, similar to that previously reported for the JEV NS2B protein. Furthermore, comparative analysis between the DENV and JEV NS2B sequences revealed striking similarities. We also observed that the overexpression of recombinant NS2B in bacteria affected bacteria cell growth and enhanced bacterial membrane permeability to hygromycin B (HygB). In addition, crosslinking tests showed the ability to form oligomers; when recombinant NS2B was incubated with erythrocyte membrane systems, it produced organized structures within the membranes, which promoted the destabilization of the erythrocyte membrane and cell lysis. Therefore, this study represents the first investigation into the potential role of NS2B in causing changes in membrane permeability during DENV infection.

## Results

### *In silico* analysis of the DENV NS2B protein identified similarity with the JEV NS2B protein

**Fig. 1:**
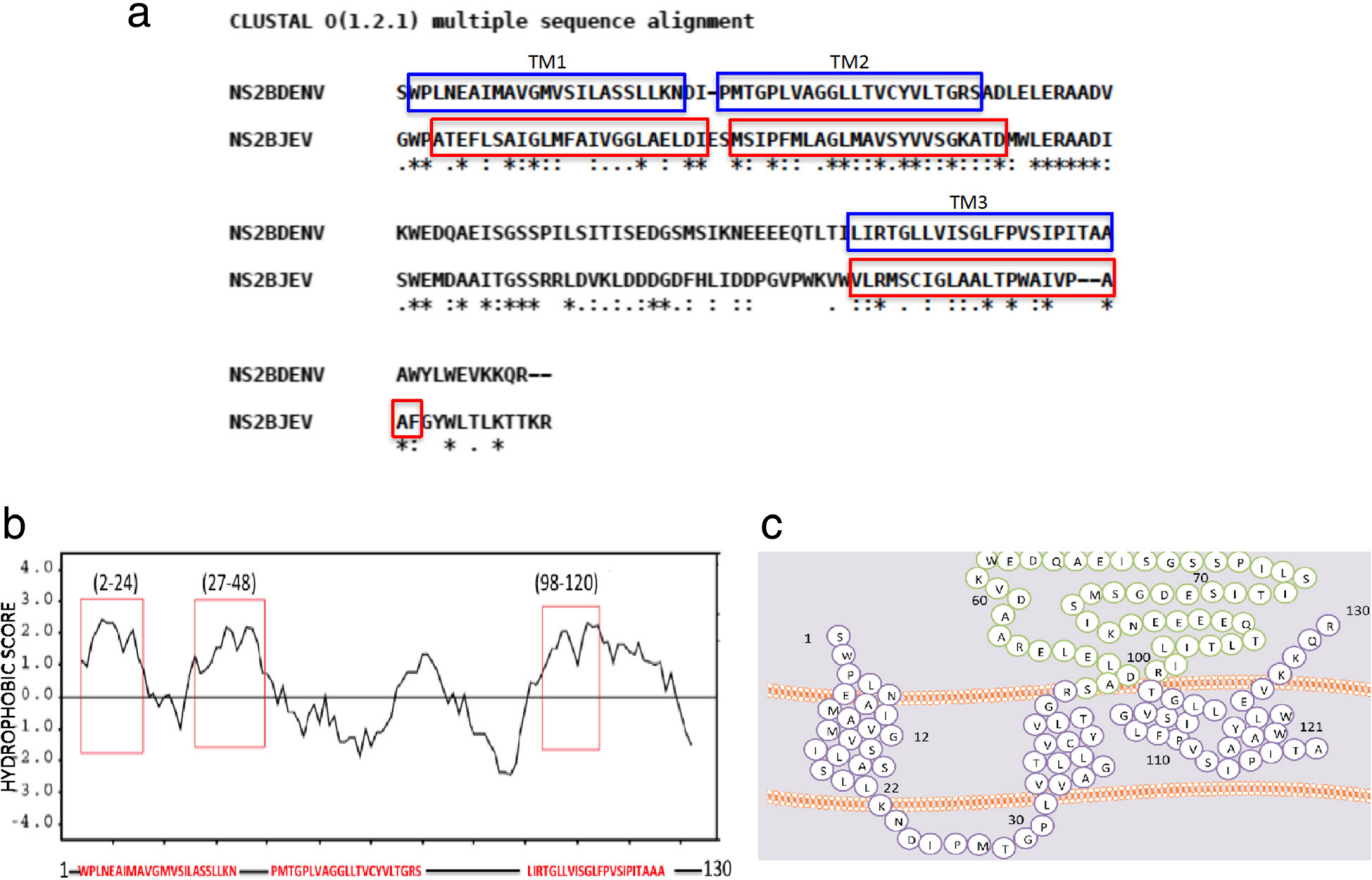
*In silico* analysis of the NS2B sequence. **a** Alignment of the NS2B sequences of DENV and JEV, using Clustal W software. The 3 transmembrane regions are indicated using blue and red boxes. Within these zones, identical (*), similar (.), and low-similarity (:) amino acids were observed. **b** Hydropathy plot of the NS2B protein generated using the Kyte–Doolittle method with SOSUI software. The red boxes delimit the transmembrane regions of the NS2B protein. **c** Proposed model of the NS2B topology, which was generated using SOCS MEMSAT software. In this model, the transmembrane segments are shown in purple, and green coloration represents the NS2B cofactor domain

Results from a previous study demonstrated that the NS2B protein from JEV could modify membrane permeability in different systems [19]. *In-silico* analysis showed a 32 % identity between the sequences of the JEV and DENV NS2B proteins. In addition, this analysis identified some amino acids within the transmembrane regions that were identical or showed strong similarity, suggesting that they maintained the biochemical proprieties (Fig. 1a). Therefore, it is likely that the membrane-altering property of both viral NS2B proteins may be conserved. Thus, we generated hydropathy plots for the NS2B proteins using the Kyte–Doolittle method. This analysis revealed 3 clear hydrophobic domains located in the amino terminal and carboxy terminus of NS2B, which may reflect the transmembrane regions present in this protein. The topology of DENV NS2B is similar to that described for JEV NS2B [19], for which 3 transmembrane regions were observed in the same positions along the amino acid sequence. In addition, aromatic and basic residues were identified in both sequences, which is an important characteristic of viral proteins with MDA (Fig. 1b). A topological representation of the NS2B protein was generated using the Socs MEMSAT program. The orientation of the 3 transmembrane regions was determined, showing that the amino and carboxyl-terminal domains were oriented towards the cytoplasm (Fig. 1c); these findings corroborate a previous report [34].

### Bacterial expression and purification of the DENV NS2B protein

**Fig. 2:**
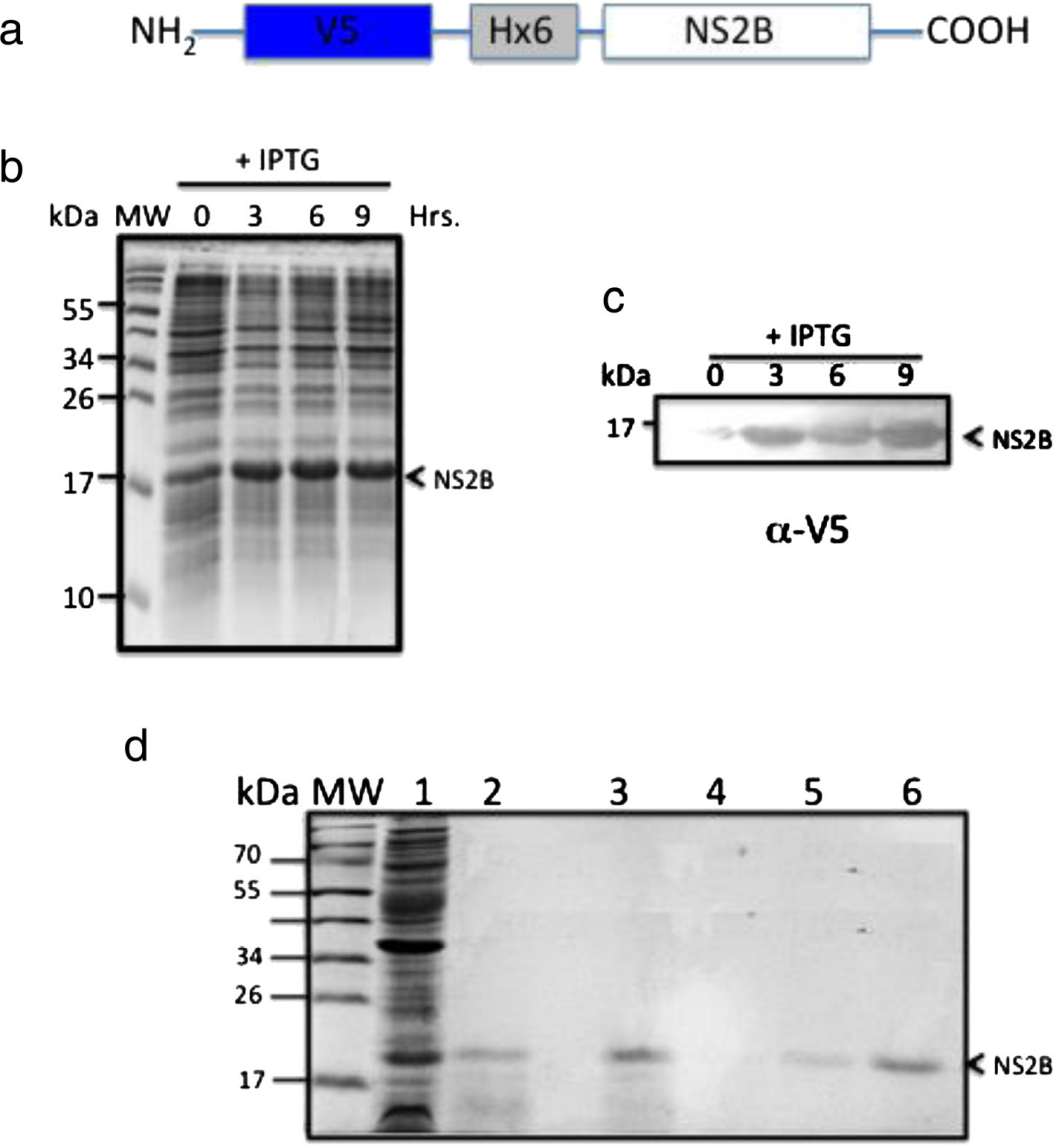
Expression and purification of NS2B protein from bacteria. **a**. Schematic representation of an expression construct with NS2B in frame with the N-terminal V5 epitope and a 6° His tag. **b** Expression and purification of NS2B protein in bacteria. Cells were induced with 1 mM IPTG at zero time and analyzed by SDS-PAGE at the indicated times post-induction. **c** Expression and identification of recombinant NS2B protein at the indicated times post-IPTG induction by western blot analysis. The NS2B protein was detected using an anti-V5 antibody at a 1:5000 dilution. **d** Analysis of recombinant NS2B protein purification following different isolation steps by SDS-PAGE and Coomassie blue. Preparative gel electrophoresis and affinity chromatography were used for purification. Lane 1: inclusion bodies purified from bacteria expressing the NS2B protein. Lane 2: fraction obtained from preparative gels, in the molecular-weight region for of a 17-kDa protein. Lane 3: proteins not bound to the resin. Lane 4: fraction washes from the resin. Lanes 5–6: fraction containing the eluted and purified NS2B protein

To obtain sufficient recombinant DENV NS2B protein for analyzing its membrane-altering activity, we cloned and expressed the NS2B protein with an N-terminus V5 epitope and a 6× His tag to facilitate its purification (Fig. 2a). This construct was used to transform *E. coli* BL21 Star cells. A clone harboring the NS2B expression vector was grown in culture and induced with IPTG 3, 6, and 9 h post-transformation (Fig. 2b). Thus, we decided to overexpress the NS2B protein. This protein was expressed in inclusion bodies, similarly to other viral proteins with viroporin activity [22, 23]. A 17-kDa band was observed by western blotting at 3, 6, and 9 h post-transformation, which was identified as the tagged NS2B protein using an anti-V5 antibody (Fig. 2c). In addition, we purified the NS2B protein using 2 strategies. The first strategy involved the induction of NS2B expression after 9 h (Fig. 2d, lane 1), which was partially purified by eluting a band of the appropriate mass (17 kDa) from a Coomassie Blue-stained gel (Fig. 2d, Lanes 2 and 3). NS2B was further purified with nickel resin via the N-terminal 6× His tag on the recombinant NS2B protein (Fig. 2d, lanes 5 and 6). Several 1 mL-aliquots were eluted from the nickel resin, and Coomassie Blue staining revealed only the presence of the 17-kDa a band in the last fraction recovered.

To demonstrate that the NS2B protein was associated with bacterial membranes, additional experiments were performed. We isolated membranes and then extracted the membrane proteins using TDPC detergent to study proteins contained in membrane fractions from inclusion bodies. The transformed bacteria were grown as described previously.

**Fig. 3:**
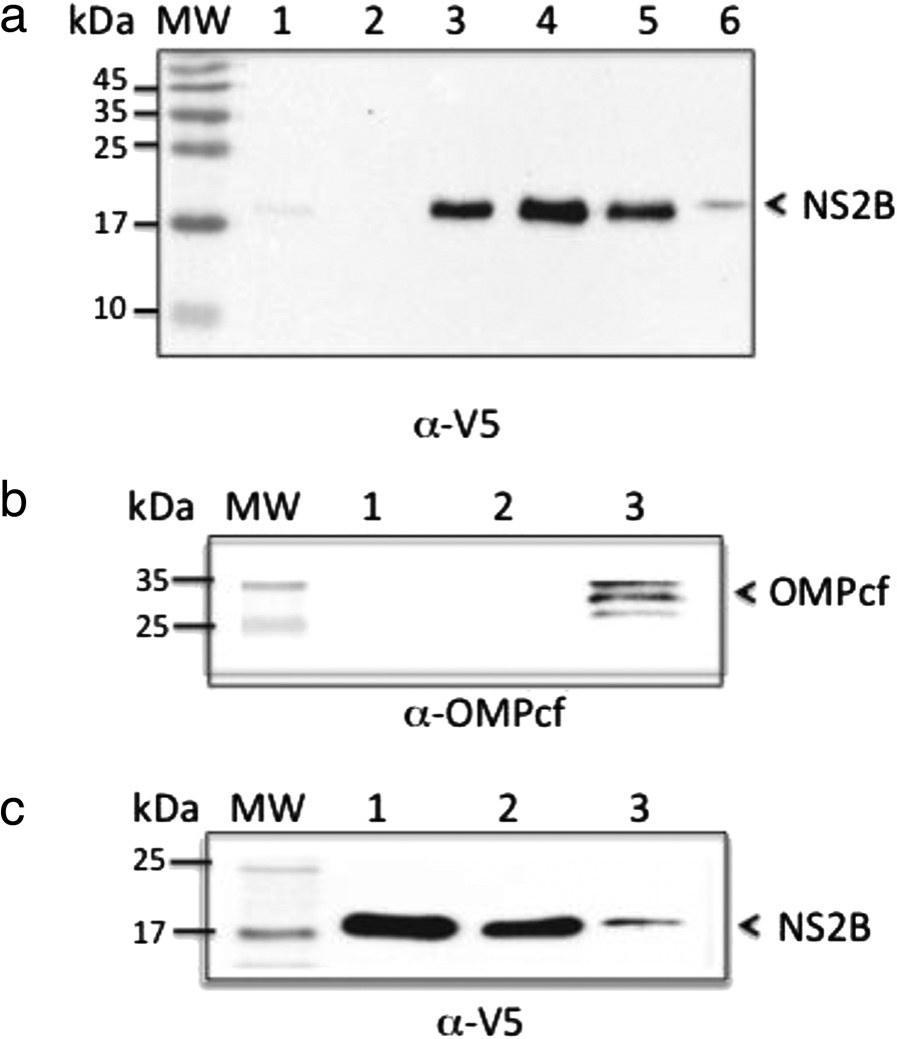
Association of the NS2B protein with bacterial membranes. **a** Western blot analysis of NS2B protein expression in *E. coli* using an anti-V5 antibody. Lane 1: NS2B clone, uninduced. Lane 2: bacteria transformed with the parental vector. Lane 3: NS2B clone, induced with IPTG. Lane 4: Inclusion bodies of bacteria transformed with NS2B. Lane 5: purified NS2B protein. Lane 6: purified bacterial membranes. **b** and **c** western blot analysis of bacterial membrane fractions using a mouse polyclonal antibodies specific to-OmpCF (resident bacterial membrane protein) and α-V5. Lanes 1 and 2: purified protein NS2B. Lane 3: bacterial membrane fraction

Next, a western blot was performed using an anti-V5 antibody to detect the NS2B protein (Fig. 3a). Subsequently, the membrane was washed, stripped and assessed by re-probed with mouse polyclonal anti-OMPF and anti-OMPC proteins (Fig. 3b and c). We found 2 bands with the predicted molecular weights of 37 kDa and 40 kDa for the porins described above respectively. This result confirmed that the NS2B protein is found in the membrane fractions with OMPC and OMPF.

### Changes in bacterial membrane permeability following expression of the NS2B protein

**Fig. 4:**
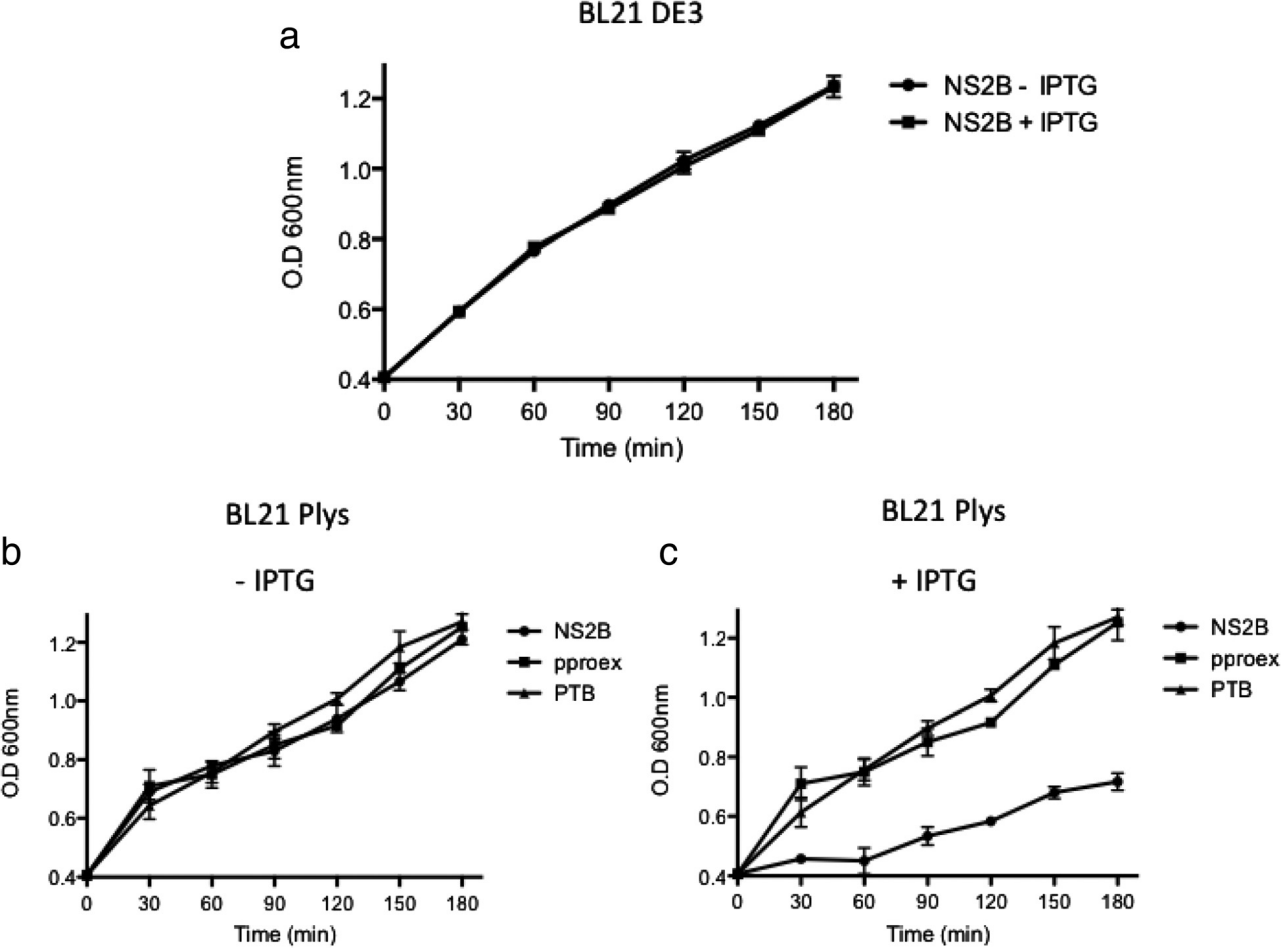
NS2B protein expression arrests *E. coli* growth and affects the permeability of bacterial membranes. **a** Grow courve of *E.coli* BL21 DE3 transformed with pProEX/NS2B (toxicity control). Growth curves of engineered *E. coli* BL21pLys transformed with NS2B, pProEX, and PTB constructs in the absence **b** or presence **c** of IPTG. The cellular density of bacterial cultures was determined every 30 min after induction by measuring optical densities at 660 nm

**Fig. 5:**
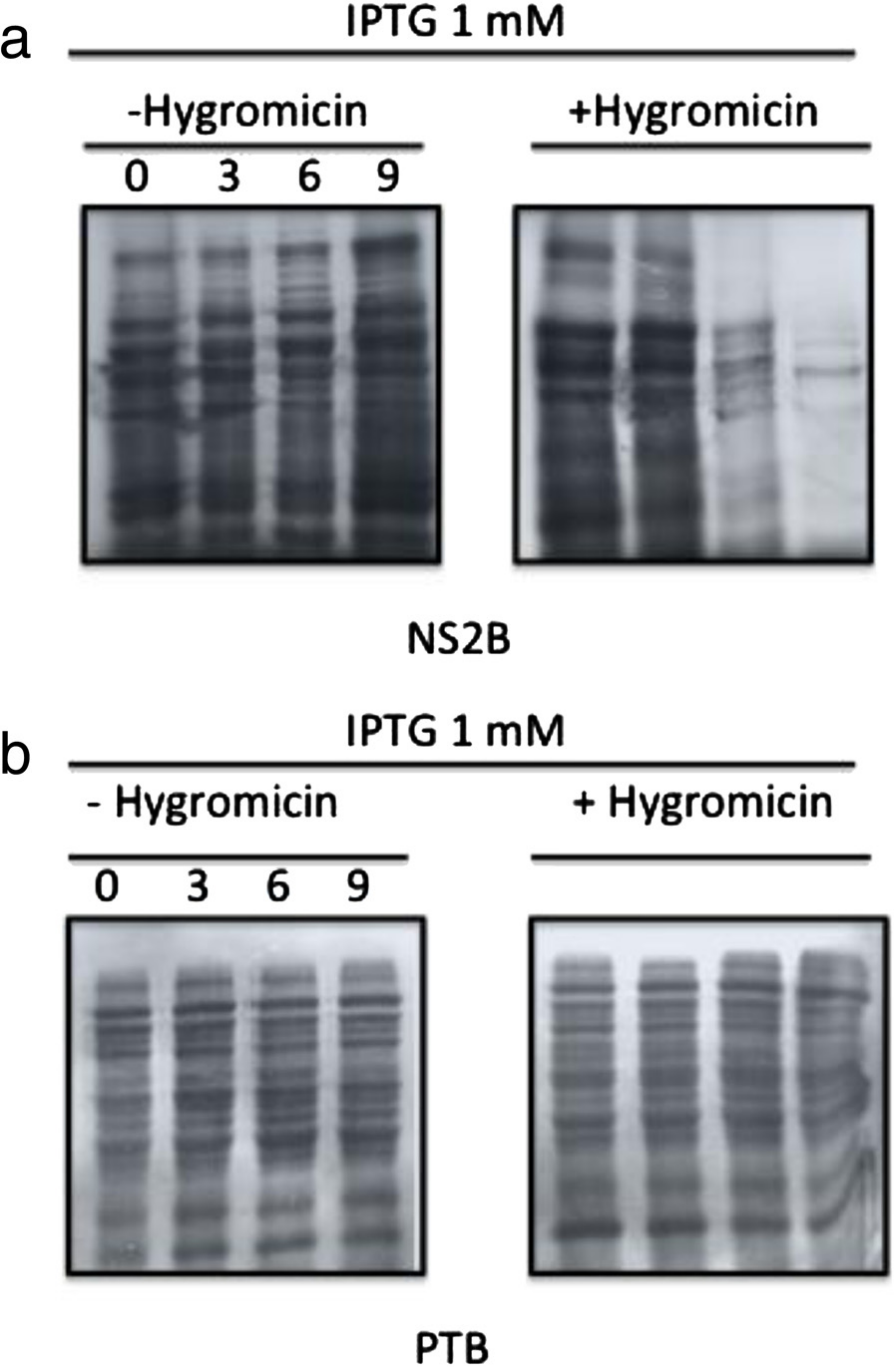
*E. coli* BL21 pLysS transformed with NS2B **a** and PTB **b** expression plasmids. Both transformant groups were induced with IPTG in the absence (−) or presence (+) of HygB. Aliquots of these cultures were subjected to metabolic labeling with [^35^S] Met-Cys at the indicated time points. The samples were resolved by SDS-PAGE and analyzed by autoradiography

*In silico* analysis suggested that the DENV NS2B protein is homologous to the JEV NS2B protein and possesses the same MDA. If the NS2B protein causes membrane destabilization, a widely known assay could be used to confirm this possibility. *E. coli* BL21 (DE3) pLysS cells, which express lysozyme, were transformed with a plasmid encoding NS2B. Disruptions in the inner bacterial membrane cause the release of the bacteriophage T7 lysozyme, which in turn promotes cell lysis that can be quantified by measuring the optical density of bacterial cultures. BL21(DE3) growth was not affected following transformation with the NS2B vector, in the presence or absence of IPTG (Fig. 4a). Bacterial growth was also unaffected in BL1(DE3)pLysS cultures transformed with the NS2B expression vector, or transformed with the parental vector pProEX in the absence of IPTG (NS2B expression not induced), when compared to negative control bacteria expressing the soluble polypyrimidine-tract-binding (PTB) protein (Fig. 4b). However, when NS2B expression was induced, bacterial growth was markedly inhibited compared to the PTB and pProEX controls (Fig. 4c). We also investigated whether NS2B expression altered the permeability of bacterial membranes, allowing entry of the translation inhibitor HygB. Thus, bacterial cultures were metabolically labeled with [S^35^]-methionine after the addition of HygB, under NS2B-inducing conditions. We observed that protein synthesis was completely inhibited in a time-dependent manner, indicating that the bacterial membranes became permeable to HygB following NS2B induction (Fig. 5a). However, no effect was observed with bacteria expressing an unrelated protein (PTB) in the absence or presence of HygB (Fig. 5b). These results strongly suggest that the NS2B protein associates with membranes, destabilizes their architecture, and allows the entrance of HygB.

### NS2B forms homo-oligomers involved in lytic effects

**Fig. 6:**
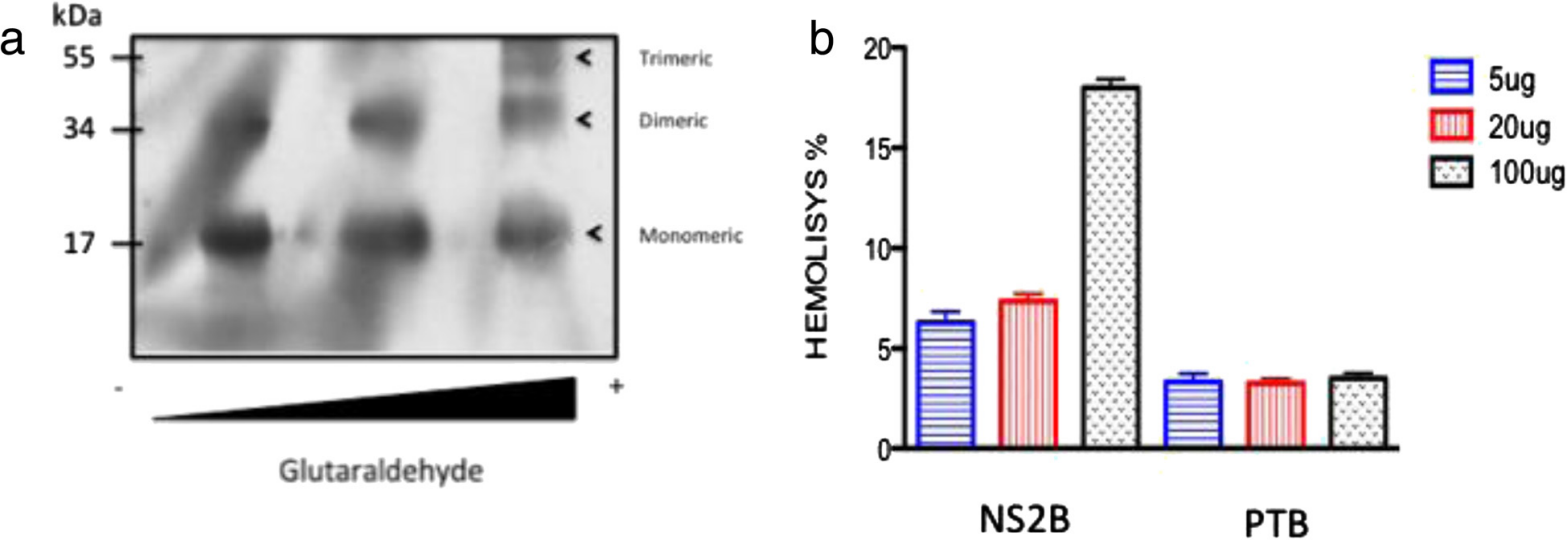
Analysis of NS2B protein to perform oligomerization. **a** The NS2B protein was incubated with different glutaraldehyde concentrations. The samples were resolved by SDS-PAGE under reducing conditions, and protein expression was analyzed by western blotting. Then, the oligomeric structures (dimers and trimers) were detected using a polyclonal antibody against the NS2B protein at a 1:3000 dilution. **b** Hemolysis assay results using human erythrocytes incubated with different concentrations of the NS2B or PTB proteins (5, 20, or 100 μg). The amount of hemoglobin released was analyzed by spectrophotometry. The percentage of lysis was calculated as indicated in the Materials and Methods section

A conserved characteristic of viroporins is the presence of hydrophobic domains that interact with lipid membranes. Another property shared by viroporins is their ability to oligomerize once inserted into cell membranes. Previous reports demonstrated that several viroporins could oligomerize in the presence of glutaraldehyde [24, 25]. To determine whether NS2B acts as a viroporin, we evaluated its ability to form homo-oligomers in vitro by chemical cross-linking. After the NS2B protein was incubated with increasing glutaraldehyde concentrations, western blots revealed the presence of 2 or 3 bands (Fig. 6a, lanes 1–3), which migrated at the expected position of monomers (17 kDa), dimers (34 kDa), and trimers (51 kDa). We hypothesized that upon membrane association of the NS2B protein, it oligomerizes to form structures similar to channel or pores that facilitate changes in membrane permeability. For this the hemolytic activity of NS2B was assayed in human erythrocytes, which are an efficient model for studying protein-membrane interactions, as well as changes in membrane permeability [26, 27] Erythrocytes were incubated at 37 °C for 1 h with various concentrations of NS2B or the negative-control PTB protein, and hemolysis levels were measured in supernatants following centrifugation. The NS2B protein showed hemolytic activity in a dose-dependent manner (Fig. 6b). Approximately 20 % of the erythrocytes were lysed when exposed to 100 μg of purified NS2B protein, while at lower quantities, the lytic activity was reduced. In contrast, hemolysis did not occur following incubation with the same concentrations of the control protein. These results suggested that the NS2B protein associated with erythrocyte membranes to form structured oligomers and caused cell lysis in a concentration-dependent manner.

### Confirmation of an organized NS2B protein structure on eukaryotic membranes

**Fig. 7:**
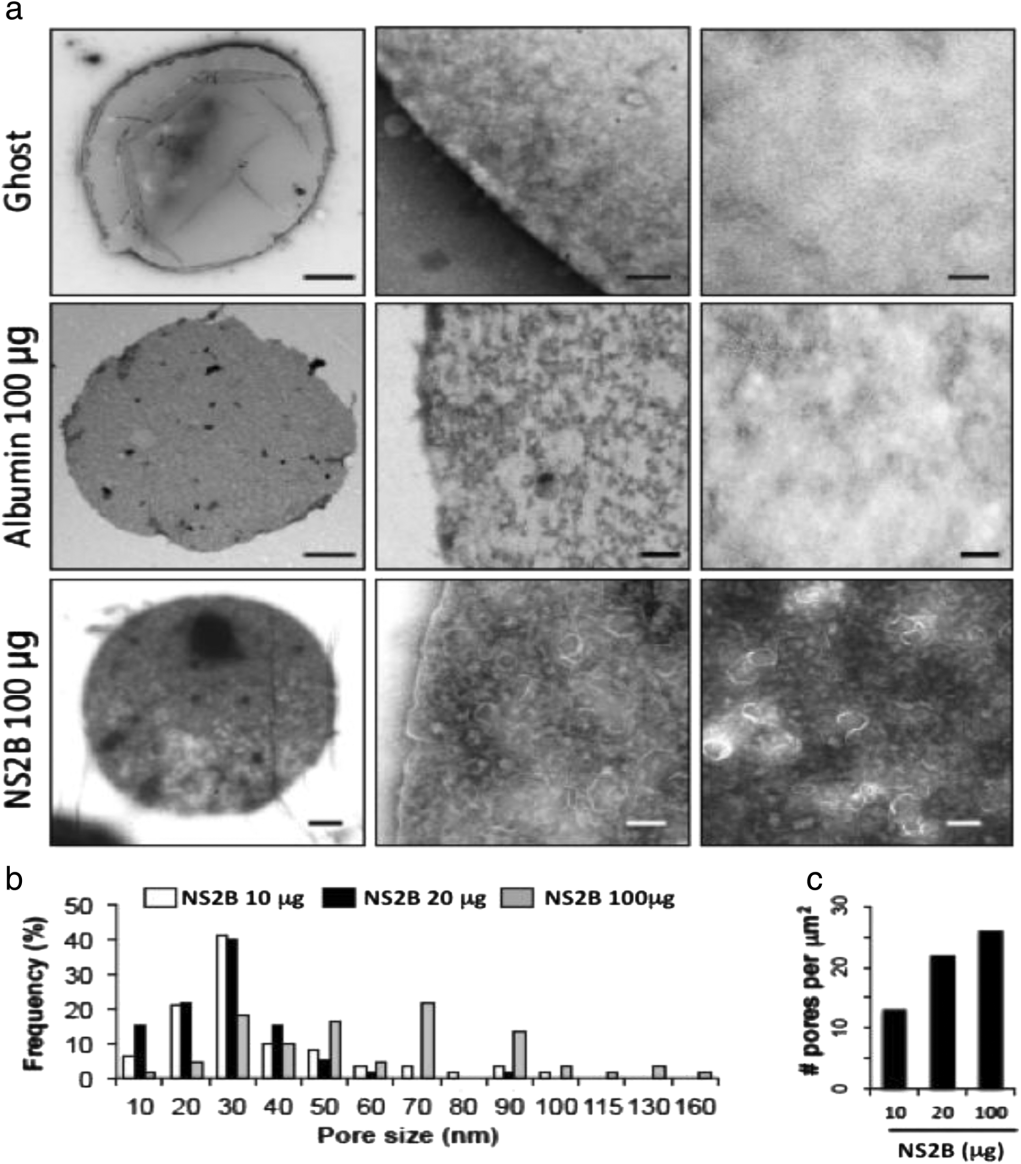
Characterization of the oligomeric structure of the NS2B protein by TEM. **a** TEM of the NS2B protein in erythrocyte ghosts. Erythrocyte membranes were purified and subsequently incubated with PBS (upper panel), albumin (middle panel or NS2B (bottom panel) protein (100 μg), or PBS. Structures with ring- or pore-like morphologies were observed in membranes incubated with the NS2B protein and were micrographed. **b** Frequency of pore size on the surface of erythrocyte ghosts using the purify NS2B protein at 10, 20 and 100 μg. **c** Quantification of number of pores on the surface of the erythrocyte ghosts at different concentrations of NS2B protein

The above results showed that NS2B formed at least trimeric structures that induced hemolytic activity. Base on this finding, we analyzed the presence of the NS2B protein in erythrocyte ghost membranes by transmission electron microscopy (TEM). This analysis revealed the presence of ring-shaped structures, the number of which was dependent on the NS2B concentration used (Fig. 7). Ring-shaped structures were not identified in erythrocytes ghosts maintained in PBS or in the presence of albumin (Fig. 7a). Analysis of these structures showed circular ring structures were also commonly associated with the membranes. These structures were similar to those observed with other viral proteins with viroporin activity. [28]. Rings with sizes between 30 and 40 nm in diameter were most frequently found, although larger structures were detected using a higher NS2B quantity (100 μg), as shown in Fig. 7b. The number of ring-shaped structures per square micron increased according the amount of NS2B used (Fig. 7c). Finally, it was confirmed that the increase in the number of ring-shaped structures at the higher NS2B quantity correlated with increased lytic activity observed in the hemolysis assays (Fig. 7c). Our results provide the first direct evidence that the NS2B protein can oligomerize in mammalian membranes and that it possesses pore-forming activity that modifies membrane permeability with lytic effects on the target membrane, as reported for other viroporins.

## Discussion

Numerous studies using different positive-strand RNA viruses have demonstrated that reorganization of the intracellular membrane can create a scaffold for the replication machinery of different viruses [29, 30]. During the *dengue* viral cycle, cell membranes are critical elements for the entrance, translation, replication, and assembly of viruses. As infection progresses, several viral proteins can disturb cell membrane structures. According to previous reports, DENV proteins promote cellular membranes reorganization, although the functions of their small hydrophobic proteins have not been studied enough in this regard. Different reports have identified a potential role that these proteins may exert on several membrane systems, such as identifying membranotropic regions, or its association with artificial membranes [31, 32]. However, it has not been determined if any of these proteins can promote changes in membrane permeability. Thus, the aim of this study was to determine if the hydrophobic NS2B protein possesses viroporin activity, considering that this protein was expressed in membranes. We and others previously showed that this protein is present in cell membranes, particularly lipid rafts [20]. A bioinformatics study performed with the NS2B sequence revealed a high degree of homology with a previously reported viroporin for a member of the *Flaviviridae* family [19]. Previous in vitro results using microsomal membranes also showed that the cleavage products NS2B and NS3 (Pro) were membrane-associated. Furthermore, this membrane requirement was due to the presence of hydrophobic regions predicted in NS2B and found in lipid rafts in the ER [20, 21].

One of more distinctive characteristics of viroporins is their ability of induce changes in membrane permeability. In this study, we provide the first evidence that pore-forming activity occurs when the NS2B protein is expressed in a bacterial system, as widely shown with other viral proteins with viroporin activity [33]. In prokaryotic systems such as *E. coli*, the NS2B protein was effective in inducing sensitivity to HygB under the conditions used. In addition, changes in permeability to HygB and alterations of bacterial growth may exert a direct effect on the incorporation of NS2B within bacterial membranes, leading to lost membrane integrity. This data are consistent with those obtained with viroporin NS2B of JEV, suggesting that the activity could be conserved.

Another important feature of viroporins is that they can oligomerize; therefore, great efforts have been taken to understand the architecture of these proteins [34, 35]. *In silico* analysis of the NS2B sequence showed the presence of 3 transmembrane regions, which may be important for membrane insertion or oligomerization. Chemical cross-linking experiments performed with glutaraldehyde revealed that NS2B could oligomerize into trimeric species under reducing conditions; however, in the absence of glutaraldehyde, dimers were also observed. The above data strongly suggest that the intermolecular associations observed do not depend on disulfide bridges. Instead, NS2B dimer and trimer formation may depend upon and hydrophobic interactions, as observed with other viroporins [24, 36]. NS2B oligomers may organize into pore structures in cell membranes, which would account for the membrane permeability alterations observed. Various systems have been employed to study these phenomena, such as dye-loaded liposomes, Xenopus oocytes, and unilamellar membranes. We employed 2 strategies to characterize these structures. In hemolysis assays, we showed that the NS2B protein in contact with human erythrocytes modified the membrane permeability, promoting the release of hemoglobin. To further address this issue, we performed TEM studies to determine whether the NS2B protein integrates into biological membranes and forms pores in the target membrane (erythrocyte ghosts). Electron micrographs showed a distribution of ring-shaped structures of variable sizes that were similar those observed for other viroporins. These ring-shaped structures increased in a dose-dependent manner with the quantity of NS2B protein added. In addition, they were absent in membrane controls maintained in PBS or in presence of albumin. These data strongly suggest that NS2B directly contacts and modifies the membrane structure.

This work provides the first direct evidence that the NS2B protein is capable of oligomerizing, producing pore-like structures in different environments, and modifying the permeability of the membrane. Future studies are necessary to assess whether the effect of the protein NS2B in these model membranes occur in the context of infection with DENV. However, it is important to mention that the protein NS2B, similar to other small hydrophobic viral proteins, is actively transported to vesicles where replication complexes are formed. The presence of pores in these vesicles has been reported [37] to facilitate the process of viral-genome packing. The origin of these pores is unknown, although it is likely that NS2B could participate in these processes.

## Conclusions

This study demonstrated the ability of the NS2B protein to function as a viroporin. Our results showed that the NS2B protein can alter the permeability of different models of membranes and that organized structures were observed in ghost erythrocyte membranes. These results suggested that NS2B potentially has MDA, a function that could be critical for steps during the DENV replication cycle.

## Methods

### DENV stock production

The stock preparation and titration of DENV-2 New Guinea strain AF0136 were performed as described previously [38]. The virus was tittered by performing standard plaque-forming assays in BHK-21 cells, as described previously. After 5 days, the resulting plaques were stained with naphthol blue-black solution and quantified [3, 39].

### Cloning of the NS2B DENV protein

The DENV-2 NS2B gene was cloned in the plasmid pET151/D-TOPO (Invitrogen). Briefly, total RNA was extracted from DENV-2-infected C6/36 cells using the Trizol reagent (Gibco, USA), according to the manufacturer’s instructions. The NS2B sequence from DENV-2 (New Guinea) was amplified by reverse transcription-PCR, using the primer pair: 5′-ctaggatccatgagctggccacta-3′ (forward) and 5′-ccggaattctcaccgttgtttcttcac-3′ (reverse). The PCR product was ligated into the pET151/D-TOPO cloning vector in frame with cDNA encoding a V5 epitope and a 6× His tag (V5H6NS2B). Finally, the plasmid from the resultant colonies was purified the next day using an EndoFree Plasmid Purification Kit (Qiagen, Inc., Chatsworth, CA). Construction was verified by automated DNA sequencing.

### Expression and purification of the NS2B protein

Bacterial cultures were transformed with V5H6NS2B, and the NS2B protein was induced for 24 h by the addition of IPTG. The bacterial lysates were then sonicated, centrifuged, and resuspended in 50 mM Tris and 10 mM EDTA. These steps were performed to pellet the inclusion bodies containing the NS2B protein, which were then analyzed by Coomassie Blue staining and western blotting with a monoclonal antibody against the V5 epitope (Invitrogen). The V5H6NS2B protein was purified by 2 methods. First, inclusion bodies were resolved by electrophoresis on a 17 % SDS-PAGE gel, and the V5H6NS2B protein was eluted from an excised gel piece in 1 % PBS overnight at 4 °C. The V5H6NS2B protein was further purified using the ProBond Purification System (Invitrogen). Briefly, nickel-coupled resin was resuspended in native-binding buffer (250 mM NaH_2_PO_4_, pH 8.0, 2.5 M NaCl) and the V5H6NS2B protein was allowed to bind the resin overnight at 4 °C. Next, the resin was washed several times with native buffer, after which the V5H6NS2B protein was eluted in elution buffer (250 mM NaHPO_4_, pH 8.0; 2.5 M NaCl; and 3 M imidazole, pH 6.0; final pH is 8.0) in 1 mL aliquots. The resulting fractions were then precipitated with boric acid, resuspended in HEPES buffer (pH 8.0), resolved by SDS-PAGE, and visualized by Coomassie Blue staining to evaluate the purity.

### Permeability assays in *E. coli* pLysS bacteria

*E. coli* BL21 (DE3) pLysS bacteria were transformed with V5H62B or pProEX-PTB and grown at 37 °C in LB medium in presence of 100 μg/ml ampicillin and 34 μg/ml chloramphenicol. When cultures reached an absorbance of 0.4, at 600 nm they bacteria cultures were induced by the addition of 1 mM isopropyl-B-D thiogalactopyranoside (IPTG). Subsequently, the optical densities were measured, and culture aliquots were taken at intervals of 30–190 min. In addition, the original cultures were diluted 100-fold in M9 medium supplemented with 0.2 % glucose and antibiotics. When the cultures reached an absorbance of 0.4, they were induced by the addition of 1 mM IPTG. Next, 1 ml aliquots of cultures were taken at the indicated times and treated with 1 mM HygB for 10 min at 37 °C. Finally, the bacteria cultures were metabolically labeled with 2 μCi/ml of [S^35^]-Met-Cys for 10 min at 37 °C, pelleted at 13,000 rpm, and resuspended in Laemmli buffer. Protein lysates were separated on a 10 % SDS polyacrylamide electrophoresis (SDS-PAGE) gel and visualized by autoradiography.

### Protein cross-linking assays

Seven micrograms of purified V5H6NS2B protein in HEPES buffer was incubated with increasing concentration (0.1–0.5 %) of glutaraldehyde (Sigma). These reactions were incubated in the dark for 2 min at 25 °C and subsequently quenched with 50 mM Tris–HCl, pH 8.0 for 15–20 min. Cross-linked proteins was separated on a 12 % SDS-PAGE gel under reducing conditions, and complexes were detected in western blots with a polyclonal anti-NS2B antibody and a secondary antibody coupled to horseradish peroxidase.

### Membrane purification

*E. coli* were harvested by centrifuging at 11,000 g for 10 min at 4 °C, the cell pellets were re-suspended in lysis buffer (20 mM Tris–HCl, pH 7.8, 300 mM NaCl, and 2 mM β-mercaptoethanol) and were then broken up by sonication on ice. The cell lysate was cleared by centrifugation at 5000 g for 20 min to remove cell debris. Then supernatant was transferred to an ultra-centrifuge tube with 4 ml of 30 % and 4 ml of 60 % sucrose solution. Cell membrane was collected from the interface after ultracentrifugation at 125,000 g for 1 h. The cell membrane fraction was collected from the interface to perform western blot experiments. [21]. A hyperimmune mouse antibody specific for OMPC and OMPF was used to re probed the membranes were NS2B protein was detected.

### Determination of hemolysis effect by NS2B protein

The hemolytic property of the V5H6NS2B protein was measured in human erythrocytes. Hemolysis assays were performed as described previously [24], with modifications. Briefly, 4 ml of whole blood was washed with PBS (pH 7.0) and centrifuged at 1500 rpm for 5 min. Next, 2 × 10^7^ blood cells were added to 3 individual tubes, to which the V5H6NS2B protein (purify by affinity chromatography as mentioned above) was added at different concentrations (5, 20, or 100 μg). Negative control experiments were performed by adding the soluble protein PTB to blood cells. In addition, 10 mL of 1 % Triton X-100 was added to blood cells as a positive control for cell lysis. The tubes were incubated at 37 °C for 1 h, centrifuged at 700 × *g* for 10 min, and the supernatants were placed in 96-well plates. The release of hemoglobin was measured spectrophotometrically at 540 nm. Specific lysis was calculated by the following formula: Specific lysis = (A_540_ lysis in the sample – A_540_ basal lysis)/(A_540_ total lysis – A_540_ basal lysis) × 100.

### Erythrocyte ghost preparation and electron microscopy experiments

To determine the organization of the V5H6NS2B protein in biological membranes, erythrocyte ghosts were prepared from blood cells. Red cells were purified from blood samples in the presence of anticoagulant and by centrifugation at 1500 rpm for 10 min, and the plasma was removed by aspiration. This procedure was performed 3 times, using 0.17 M NaCl buffer to re-suspend cells following each centrifugation step. After the third centrifugation step, the pelleted cells were resuspended in 0.17 M NaCl buffer to the original sample volume. Next, 1 mL samples were hemolyzed by incubation with 6 mL of buffer (1 mM EDTA, 9.64 mM NaCl, 3.61 mM Na_2_HPO_4_, and KH_2_PO_4_ and KH2PO4 at 1.20 mM; pH 7.2) on ice for 20 min. The lysates were then centrifuged at 17,900 × *g* at 4 °C for 10 min, the supernatants were removed, and the pellet was washed sequentially in 9.6 mm Tris–HCl and 4 mM NaCl (pH 7.2) and a buffer containing 4.8 mm Tris–HCl, 10 mM NaCl, and 100 mM KCl (pH 7.2). Finally, the erythrocyte ghost pellets were washed twice in water and re-suspended in 1 mL of HEPES buffer. For microscopic analysis of incorporation of NS2B protein into membranes, 15 μl of V5H6NS2B protein (100 μg/mL) or bovine serum albumin (negative control) were diluted in HEPES buffer (pH 7.0). The diluted V5H6NS2B or bovine serum albumin proteins were mixed with 100 μl of erythrocyte ghosts and incubated for 1 h at 37 °C. Following the incubation period, the samples were washed in HEPES buffer and centrifuged at 1376 × *g* for 5 min. Twenty-microliter samples were then applied to copper grids previously coated with a thin layer of poly[vinyformval] (Polysciences, Inc., Warrington, PA). Excess sample was drained with a filter paper and negatively stained with a solution of 2 % uranyl acetate for 30 s. Grids containing the erythrocyte ghosts were drained with a filter paper and dried at room temperature. Samples were analyzed in a JEOL 1400X transmission electron microscope at 80 ke (JEOL, Ltd., Japan).
